# Interrelationship between daily COVID-19 cases and average temperature as well as relative humidity in Germany

**DOI:** 10.1038/s41598-021-90873-5

**Published:** 2021-05-28

**Authors:** Naleen Chaminda Ganegoda, Karunia Putra Wijaya, Miracle Amadi, K. K. W. Hasitha Erandi, Dipo Aldila

**Affiliations:** 1 Department of Mathematics, University of Sri Jayewardenepura, Nugegoda, 10250 Sri Lanka; 2 Mathematical Institute, University of Koblenz, 56070 Koblenz, Germany; 3 Department of Mathematics and Physics, Lappeenranta University of Technology, 53851 Lappeenranta, Finland; 4 Department of Mathematics, University of Colombo, Colombo, 00300 Sri Lanka; 5 Department of Mathematics, Universitas Indonesia, Depok, 16424 Indonesia

**Keywords:** Influenza virus, Applied mathematics, Statistics

## Abstract

COVID-19 pandemic continues to obstruct social lives and the world economy other than questioning the healthcare capacity of many countries. Weather components recently came to notice as the northern hemisphere was hit by escalated incidence in winter. This study investigated the association between COVID-19 cases and two components, average temperature and relative humidity, in the 16 states of Germany. Three main approaches were carried out in this study, namely temporal correlation, spatial auto-correlation, and clustering-integrated panel regression. It is claimed that the daily COVID-19 cases correlate negatively with the average temperature and positively with the average relative humidity. To extract the spatial auto-correlation, both global Moran’s ${\mathscr {I}}$ and global Geary’s ${\mathscr {C}}$ were used whereby no significant difference in the results was observed. It is evident that randomness overwhelms the spatial pattern in all the states for most of the observations, except in recent observations where either local clusters or dispersion occurred. This is further supported by Moran’s scatter plot, where states’ dynamics to and fro cold and hot spots are identified, rendering a traveling-related early warning system. A random-effects model was used in the sense of case-weather regression including incidence clustering. Our task is to perceive which ranges of the incidence that are well predicted by the existing weather components rather than seeing which ranges of the weather components predicting the incidence. The proposed clustering-integrated model associated with optimal barriers articulates the data well whereby weather components outperform lag incidence cases in the prediction. Practical implications based on marginal effects follow posterior to model diagnostics.

## Introduction

Viral diseases emerge with complex transmission dynamics, and they are hard to eradicate challenging capacity of testing, diagnosis, and cure^1,2^. Such complexity is generated by various factors such as genetic changes of the virus, environmental influences, and host behavior^3,4^. COVID-19 caused by the coronavirus SARS-CoV-2 has also shown its revolutionary dynamics via all those routes, leaving the world at a standstill in many aspects. The transmission of coronavirus occurs and escalates in diverse means. Most notable drivers include direct contact with infectious individuals^5^, fomite transmission via contaminated surfaces^6,7^, transmission via virus-carrying aerosols^8,9^, congested living and mobility leading to superspreading events^10–13^, and lack of compliance to health guidelines^14–17^. Though both direct and indirect transmission are recognized, the influence of outdoor aerosol transmission is not properly understood^18,19^. Meanwhile, within-household is much higher compared to cross-household transmission leaving home quarantine also at risk^20^. Thus, planning healthcare and interventions has also become challenging. It is further problematic due to the presence of asymptomatic cases^21^.

Transmission and morbidity of COVID-19 can be worsened when co-infections with other respiratory viruses are present. Several clinical studies from different countries have observed the co-infection of COVID-19 with other viral infections^22–24^. The most common respiratory viruses are influenza virus, respiratory syncytial virus, parainfluenza viruses, metapneumovirus, rhinovirus, adenoviruses, bocaviruses, and coronaviruses^25^. These viral infections share common symptoms such as sneezing, cough, sore throats, and fever while following similar ways of transmission^26,27^. Influenza viruses that cause seasonal flu would easily co-exist with COVID-19 in the winter season^28^. This is motivated by the fact that most respiratory pathogens are seasonal^29,30^. Thus, given that many COVID-19 infected cases are undetected^31^, sneezing and cough due to another infection may allow passing respiratory droplets carrying SARS-CoV-2 too. Although the information is still limited, one cannot set aside the possible risk of excessive COVID-19 spread due to co-infection^32,33^. In this regard, timely detection is important to curtail issues of missed diagnoses^34^.

The influence of weather components such as temperature and relative humidity on the transmission of SARS-CoV-2 is investigated recently. Related studies have been motivated by the fact that temperature and relative humidity also regulated the survival of coronaviruses of SARS^35–38^ and MERS^39,40^. Respiratory droplets play a key role in transmission, subsequently more structured with aerosols and fomites^41,42^. Due to other confounding factors related to specific geographical areas, mixed findings can be expected with different levels of temperature and relative humidity^43–46^. Using panel regressions, a study of 20 countries having the most number of confirmed cases^47^ suggested that high temperature and relative humidity reduce transmission, while low temperatures are contributory for activation and infectivity of the virus. A low temperature range (− 6.28 $^{\circ }$C to + 14.51 $^{\circ }$C) has been identified as favorable to COVID-19 growth in^48^ via a statistical estimation. This study also found that a 1 $^{\circ }$C rise in temperature can reduce the number of cases by 13–17 per day. On the contrary, a study covering many cities in China^49^ using a generalized additive model found no evidence supporting the decrease in the number of cases in warmer weather. Moreover, an SEIR model calibrated for 202 locations in 8 countries^50^ showed no significant changes in the number of COVID-19 confirmed cases with a broad range of meteorological conditions. Another study in New South Wales, Australia^51^, revealed a weak correlation between COVID-19 cases and temperature, but a negative correlation between cases and relative humidity. Studies using data for the earlier infections in Jakarta with average temperature (26.1–28.6 $^{\circ }$C)^52^ and Bangladesh with average temperature (23.6–31.1 $^{\circ }$C) and minimum temperature (17.3–29.3 $^{\circ }$C)^45^ indicated significantly positive correlation. In addition, COVID-19 cases in China showed negative correlations with both temperature and relative humidity as investigated in^53^ while those in 190 countries revealed non-linear correlations with both daily temperature and relative humidity as in^54^. In Iran, also according to^55^, there was no clear evidence to relate the number of confirmed cases with warm or cold weather in different provinces, leaving population size to be a determinant factor. A related study for India was carried out using minimum temperature, maximum temperature, average temperature, and specific humidity (ratio of the mass of water vapor to the total mass of the air parcel) as the weather components^56^. The results showed a high positive correlation between COVID-19 cases and temperature measures and a low positive correlation between COVID-19 cases and specific humidity. In Germany, the confirmed cases hit 17 million by the first week of January 2021. The second wave escalation began in autumn and continued in winter. Daily cases exceed 20,000 in many days at the latter stage, where it was over 15,000 for other days in the last two months of 2020. The long-standing plateau of total deaths has also altered since November to a sharp increase and reached 35,000 at the beginning of 2021.

Motivated by the increase of morbidity during autumn and winter, this study employed panel COVID-19 incidence data from Germany and scrutinized their relationship with weather data. In some studies, weather components like temperature were collected in categories such as average, maximum, and minimum level^52,56–58^, while others used daily average extracted on a defined regular interval^50,59^. Furthermore, in some other studies, either absolute humidity^59,60^ or specific humidity^56^ was employed instead of relative humidity. Ours utilized the average of daily average temperature and relative humidity from January 31, 2020 to December 15, 2020, from three representative weather stations in Germany. Besides data availability and similarity with other studies^61,62^, the rationale behind the choice of the weather components lies in their readability throughout academia and the fact that no prior and posterior transformation are needed to obtain marginal effects. Extensive investigation on Moran’s ${\mathscr {I}}$ and Geary’s ${\mathscr {C}}$ statistics then followed so as to cover spatial auto-correlation and related practical implications. The difference with previous studies is that the temporal progression of the statistics is presented. Subsequently, this study brought forward a random-effects model with a clustering strategy. Our holistic idea lies in which ranges of the incidence are well predicted by the weather components. This is somewhat contrasting to determining the ranges of the weather components that can predict the incidence. Our clustering is based on the method of stratifying incidence data into an arbitrary number of clusters, separated by barriers. The temperature and relative humidity data were also grouped corresponding to the clustered incidence data. This not only improves fitting by providing more explanatory variables but also screens incidence clusters where the weather components fail to predict. Relevant implications using marginal effects for sample cases then followed posterior to model diagnostics.

**Figure 1:**
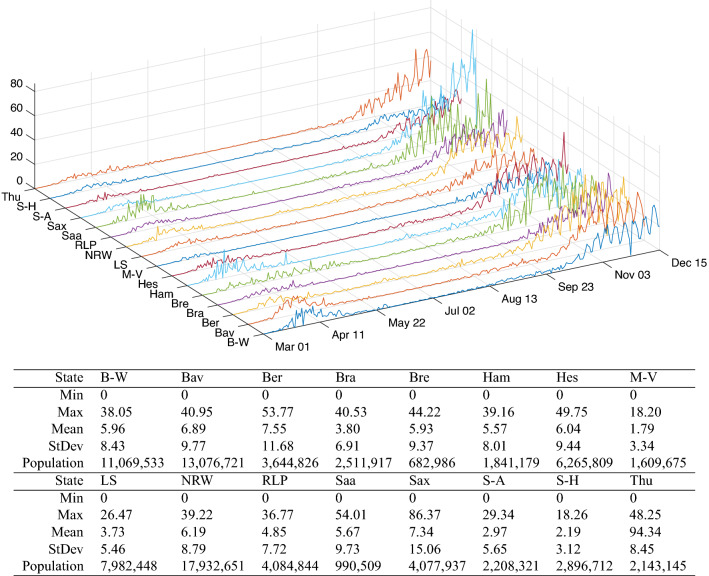
Daily COVID-19 cases per 100,000 inhabitants from all 16 states in Germany from March 01 until December 15, 2020: B-W (Baden-Württemberg), Bav (Bavaria), Ber (Berlin), Bra (Brandenburg), Bre (Bremen), Ham (Hamburg), Hes (Hesse), M-V (Mecklenburg-Vorpommern), LS (Lower Saxony), NRW (North Rhine-Westphalia), RLP (Rhineland-Palatinate), Saa (Saarland), Sax (Saxony), S-A (Saxony-Anhalt), S-H (Schleswig-Holstein), Thu (Thuringia). Population data come from the 2018 census by the Federal Statistical Office of Germany^63^.

**Figure 2:**
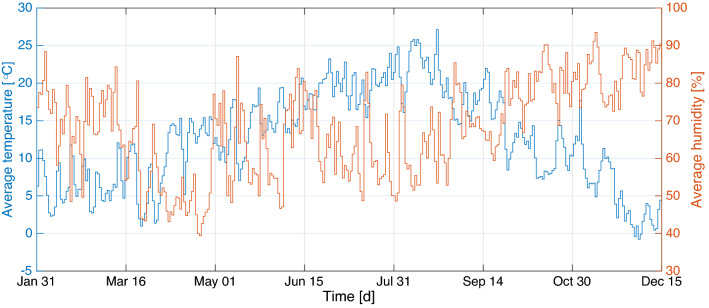
Average from the daily average temperature and relative humidity from the three weather stations in Germany: Berlin-Marzahn (Berlin), München-Stadt (Bavaria), Stuttgart-Schnarrenberg (Baden-Württemberg). Time window spans from January 31 until December 15, 2020. The tuples (Min, Max, StDev) are given by (− 0.766 $^{\circ }$C, 27.13 $^{\circ }$C, 6.45 $^{\circ }$C) for the temperature and (39.38%, 93.53%, 12.71%) for the relative humidity, respectively.

## Data and methods

### COVID-19 and weather situation in Germany

According to the official 2018 census, the German states considerably vary in population, with North Rhine-Westphalia and Bremen having the highest and lowest population size of about 17,932,651 and 682,986, respectively, out of the total population size of 83,019,213. The states also have varied economic capacities in business, industries, tourism, and education, which affect their population size. For instance, the largely populated states like Bavaria and Baden-Württemberg have booming economy and offer plenty of employment opportunities due to the situation of renowned business centers and industries, whereas low-populated states e.g. Bremen are laid behind (see^64,65^). Apparently, the number of cases and fatalities relatively depends on the population size. For instance, based on the report from Robert Koch Institute (RKI) on December 16, 2020, the largest populated state shared the highest 7-day incidence cases, and the smallest populated state shared the lowest. Given that the cases are population-driven, the dataset used for this study includes the daily confirmed COVID-19 cases for all the states from the official website of RKI^66^, which was later normalized per 100,000 inhabitants using the 2018 population census, see Fig. 1. This dataset spans the time window from March 01, 2020 to December 15, 2020. The normalization was intentional toward making the number of cases comparable across the states so as to allow for appropriate comparison with weather components that do not depend on the population (see similar treatments in^59,67,68^). Here, the daily cases were defined as the difference of the confirmed cases since the earliest time of the report. As for the accompanying weather components, temperature and relative humidity data were retrieved from climate environment open data^69^. Time series of average temperature and relative humidity were obtained using the records of three weather stations Berlin-Marzahn (Berlin), München-Stadt (Bavaria) and Stuttgart-Schnarrenberg (Baden-Württemberg). This choice was motivated by data availability and the fact that the weather pattern throughout Germany is more or less the same, except in the alps where a negligible percentage of humans live. Average temperature ranges from − 0.766 to 27.13, and average relative humidity ranges from 39.38 to 93.53%. It seems the two weather components have a negative correlation showing equivalence between low temperature and high relative humidity or vice versa. Moreover, looking at the plot of cases by month in Fig. 1 in comparison with the weather components in Fig. 2, it can be seen that cases are generally higher in colder season and considerably reduce during the hot season.

**Figure 3:**
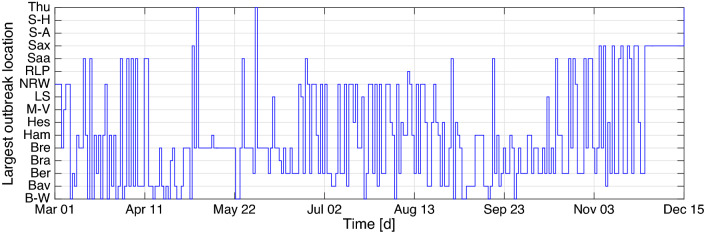
Spatial concurrence of the largest outbreak.

In addition to the reported incidence, the spatial movement of the largest outbreak over the 16 states is also worth investigating. As depicted in Fig. 3, several stages in the timeline can be identified according to the dominance shown by different states. In the first three weeks in March, the largest incidence mainly altered between Hamburg and Baden-Württemberg. Bavaria and Saarland replaced them in the next three weeks. Bavaria hold a local election on March 15, and in the next day, a state of emergency was declared for 14 days with mobility restrictions^70^. Moreover, it is the first state to declare curfew that was imposed on March 20^71^. Saarland neighboring with badly affected French region Grand Est also incurred the same situation at midnight on the same day^72^. Lack of protective clothing and closure of medical practices were also reported from Bavaria^73^. Thus, Bavaria owed the largest incidence from time to time, even after the first few weeks. Outbreaks in initial reception facilities also contributed to the increase of cases in Bavaria. The largest incidence in May and in the first two weeks of June was dominated by Bremen. It was followed by Berlin and North Rhine-Westphalia until the end of August. A sudden increase of cases was reported in North Rhine-Westphalia due to proactive case tracing, in particular at a meat factory in Coesfeld^74^. Later another cluster occurred on June 17 in a slaughterhouse in Gütersloh, North Rhine-Westphalia, leaving superspreading the main cause of spread^75^. Hamburg and Bremen also came to notice in September and October. The latter stage of October was dominated by Saarland and Berlin. In November, the largest incidence altered between Saxony and Berlin, while Saxony kept the dominance for the first two weeks of December. Saxony had shown early signs of vulnerability, prohibiting residents from leaving their dwellings similar to Bavaria and Saarland. Berlin prevailed as the most responsible state in the latter two-third of the timeline. A large-scale protest was held on August 1 in Berlin against preventive measures. This hints lack of compliance to wearing face masks and keeping physical distance that supports increasing incidence^76^.

### Correlation studies

Referred studies in “Introduction” illustrate how meteorological factors correlated with the transmission of COVID-19. Highly transmissible disease like COVID-19 requires pathogens to remain active outside of the host body and relative humidity and temperature affect the virus’s survival in the environment^44,77^. Another study engineering a SARS-CoV-2 isolate came across the fact that the virus can survive at least 28 days at ambient temperature 20 $^{\circ }$C and 50% relative humidity on non-porous surfaces and is sensible to the variation of the weather components^78^. Therefore, it is considered noteworthy to examine the interrelationship between COVID-19 cases and meteorological factors. Many statistical methods have been used in earlier studies. According to the recent review in^61^, applicable methods other than descriptive analysis are Pearson correlation coefficient, linear, and non-linear regression, LOESS, two-way ANOVA, etc. Wavelet coherency analysis was used in^50^. This study used the Spearman-rank correlation so as to evaluate both the linear and monotonic relationship between two tested covariates. Additionally, auto-correlation between reported COVID-19 cases was also done by piling the spatiotemporal data into one time series, considering that normalized data vary in relatively small numbers. Lags up to 7 days from presently were selected. Therefore, every covariate augments 16 times 283 observations where the lag-0 time series consists of time window from March 8, 2020 to December 15, 2020. Both the Pearson and Spearman-rank correlation coefficients were computed.

### Spatial pattern

Of special interest in this study is the degree of interconnection between all states in raising or decreasing the number of cases. The global Moran’s ${\mathscr {I}}$^79^ in comparison with the global Geary’s ${\mathscr {C}}$^80,81^ and its local decomposition known as Moran’s scatter plot were used. The global measures serve to indicate the overall correlation between daily COVID-19 cases per 100,000 inhabitants in every state with the weighted average of the cases in neighboring states, which refers to the *spatial lag* of the state^82^. The spatial pattern is commonly seen to lie between three extreme cases: *locally clustered*, *random*, and *locally dispersed*. Locally clustered refers to the situation where neighboring states are similar in the level of daily new cases, under which spatial dependency rules out the spatial pattern. Locally dispersed refers to the inverse spatial dependency where neighboring states are dissimilar. Something in between is then referred to as random. Representation of these spatial patterns can be understood with the aid of a chessboard. If the spatial profile of daily cases in all states resembles the chessboard, then the spatial pattern is completely locally dispersed. If all the black cells would have gathered in one spot, then the spatial pattern is completely locally clustered. The random spatial pattern is then recognized from the way the black and white cells locate randomly on the board. This is extreme binary stratification that could never occur in the realism of epidemics, from which the corresponding global measure rarely reaches its bounds.

Let us suppose that time is fixed and the daily cases from all states are reported as $C=(c_1,\ldots ,c_{S})^{\top }$ with mean ${\bar{c}}$. The other main ingredient in spatial auto-correlation is the spatial weight matrix $W=(w_{ij})$, which measures the degree of contiguity among all the states. This study used the binary adjacency matrix, where $w_{ij}$ is 1 in case *i* and *j* share a common border or 0 in case otherwise (including diagonal entries). This definition is commonly used in the literature (referred to as “queen case”) in contrast to distance-based proximity measure where central locations play a significant role as well as a definition of being a “center” is required to define the distances. Let us write $Z=(z_1,\ldots ,z_S)^{\top }:=C-{\bar{c}}$ and define $|W|:=\sum _{i,j}w_{ij}$. The global Moran’s ${\mathscr {I}}$ and Geary’s ${\mathscr {C}}$ statistic are given by

$$\begin{aligned} {\mathscr {I}}:=\frac{S}{|W|}\cdot \frac{Z^{\top }WZ}{Z^{\top }Z}\quad \text {and}\quad {\mathscr {C}}:= \frac{S-1}{2|W|}\cdot \frac{\sum _{i,j}w_{ij}(c_i-c_j)^2}{Z^{\top }Z} \end{aligned}$$

respectively. According to the formulas, the global Moran’s ${\mathscr {I}}$ represents the standardized spatial autocovariance by the variance of the data, while the global Geary’s ${\mathscr {C}}$ replaces the autocovariance by the sum of the squared differences in all data values. Both formulas then differ in sensitivity controlled by the autocovariance. In terms of stability against uncertainty in the data, Wijaya et al. in^68^ describe how Geary’s ${\mathscr {C}}$ tends to vary less significantly than Moran’s ${\mathscr {I}}$ when data are perturbed using noise of any kind. The current study presented Geary’s ${\mathscr {C}}$ only for the sake of comparison. A measurement $0<{\mathscr {I}}\rightarrow 1$ (similarly $1>{\mathscr {C}}\rightarrow 0$) indicates the direction toward locally structured spatial pattern; ${\mathscr {I}}=0$ (or ${\mathscr {C}}=1$) random spatial pattern; and $0>{\mathscr {I}}\rightarrow -1$ (or $1<{\mathscr {C}}\rightarrow 2$) locally dispersed spatial pattern. Statistical inference is usually done under a total randomization assumption to have a decision outcome based on the values of the statistics^83^. The p-value is generated after normalization using the expected values ${\mathbb {E}}({\mathscr {I}})=-1/(S-1)$, ${\mathbb {E}}({\mathscr {C}})=1$ and variances ${\mathbb {V}}({\mathscr {I}})$, ${\mathbb {V}}({\mathscr {C}})$ reported in the original studies^79,80^. The null hypothesis is that there is no spatial auto-correlation of the daily cases on the observed *S* states, meaning that ${\mathscr {I}}\simeq {\mathbb {E}}({\mathscr {I}})$ and ${\mathscr {C}}\simeq {\mathbb {E}}({\mathscr {C}})$. Therefore, a p-value smaller than a predefined significance level $\alpha$ rejects the null hypothesis whereby either a locally structured or a locally dispersed spatial pattern occurs.

In contrast to the global measures, Moran’s scatter plot measures the extent to which a state is considered a “hot spot” or “cold spot” or something in between^83^. It reports the coordinates $\left( Z/\sigma _C, W Z/\sigma _C\right)$ for all states, with $\sigma _C=\sqrt{Z^{\top }Z/S}$ denoting the standard deviation of *C*. As a row-standardized weight matrix is utilized, i.e., $|W|=S$, the pooled estimator of the regressing linear line for these coordinates passing through the origin is given by $(0,{\mathscr {I}})$. In the present context, a hot spot is defined as a state with a large number of daily cases surrounded by those with large numbers of cases (*high-high*). In the 2-dimensional Euclidean space, the coordinates of hot spots locate in the upper-right quadrant Q1. A cold spot, on the contrary, defines a state with a small number of cases surrounded by those with small numbers of cases (*low-low*). The coordinates of cold spots gather in the lower-left quadrant Q3. Other than these, local dispersion may occur falling into the following categories: a state with a small number of cases surrounded by those with large numbers (*low-high*) in the upper-left quadrant Q2, and a state with a large number of cases surrounded by those with small numbers (*high-low*) in the lower-right quadrant Q4. From the practical point of view, being a hot spot or cold spot may only rely on the health care capacity to ameliorate the disease burdens without imposing further restrictions to travel around neighboring states, except for those who travel across the border between scattered hot spots and cold spots. A state in a high-low or low-high spatial pattern, however, requires more restriction in traveling to neighboring states as the disease may diffuse (in case of high-low) or be absorbed (in case of low-high).

### Simple case–weather relation

**Figure 4:**
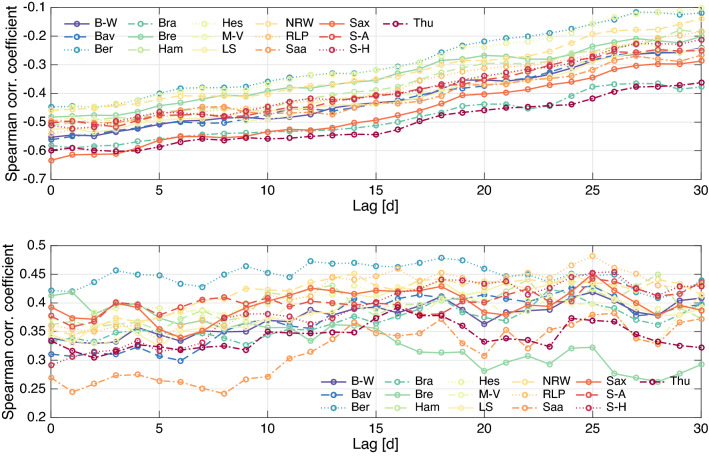
Spearman-rank correlation coefficients between daily cases from all states in Germany with the average temperature (above) and average humidity (below) on a moving window of 290 observations. Averaging throughout the states obtains the minimum of − 0.5223 (temperature) and maximum of 0.4194 (humidity) corresponding to the lags 0 and 25, respectively.

Let *i* and *j* denote the state and time index where $i\in \{1,\ldots ,S=16\}$ and $j\in \{1,\ldots ,N\}$. Our approach to modeling daily COVID-19 cases in all states in Germany was based on directly relating collected entities. These include presently (lag-0) reported cases $C:=(c_{ij})$, cases reported on the past seven days (lag-1, $\ldots$, lag-7) from presently $C_{-1}:=(c_{i,j-1}),\ldots ,C_{-7}:=(c_{i,j-7})$, average air temperature $T:={\mathbbm {1}}_{S}\otimes (t_{j})$, and lag average relative humidity $H:={\mathbbm {1}}_{S}\otimes (h_{j-25})$ corresponding to the cross-correlation result in Fig. 4. The notations ${\mathbbm {1}}_{S}$ and $\otimes$ denote the column vector of size *S* whose entries are 1 and the Kronecker product between two matrices, respectively. The final size of our observations is the entire time window length minus the maximal autoregressive lag, which is $N:=290-7=283$ (i.e. from March 8 until December 15, 2020). Let us denote $\beta _0$ as the intercept, $\beta _{\text {ind}}:=(\beta _1,\ldots ,\beta _{S-1})$ as the individual-specific effects (cut down by one term to avoid linear dependence with the intercept), $\beta _{-i}$ (for $i=1,\ldots ,7$) as the marginal effects of the lag incidence cases, $\beta _T$ as the marginal effect of the temperature, $\beta _H$ as the marginal effect of the relative humidity, and $\varepsilon =(\varepsilon _{ij})$ as the idiosyncratic error. The direct relationship among these covariates intends to not only skip additional transformations but also return direct marginal effects represented by the coefficients of the corresponding explanatory variables. This reads as

1 $$\begin{aligned} C = \beta _0{\mathbbm {1}}_{S\times N} + \sigma ^{(0)}{\mathbbm {1}}_{N}^{\top }\otimes [\beta _{\text {ind}} \,0]^{\top } + \sum _{i=1}^7\sigma ^{(i)}\beta _{-i}C_{-i}+ \sigma ^{(8)}\beta _TT+\sigma ^{(9)}\beta _HH+\varepsilon , \end{aligned}$$

which folds

$$\begin{aligned} \begin{pmatrix} c_{11} &{}\quad \cdots &{}\quad c_{1N}\\ \vdots &{}\quad \ddots &{}\quad \vdots \\ c_{S1} &{}\quad \cdots &{}\quad c_{SN} \end{pmatrix}&= \begin{pmatrix} \beta _0 &{}\quad \cdots &{}\quad \beta _0\\ \vdots &{}\quad \ddots &{}\quad \vdots \\ \beta _0 &{}\quad \cdots &{}\quad \beta _0 \end{pmatrix} + \sigma ^{(0)}\begin{pmatrix} \beta _1 &{}\quad \cdots &{}\quad \beta _1\\ \vdots &{}\quad \ddots &{}\quad \vdots \\ \beta _{S-1} &{}\quad \cdots &{}\quad \beta _{S-1}\\ 0 &{}\quad \cdots &{}\quad 0\\ \end{pmatrix}+\sum _{i=1}^7\sigma ^{(i)}\beta _{-i}\begin{pmatrix} c_{1,1-i} &{}\quad \cdots &{}\quad c_{1,N-i}\\ \vdots &{}\quad \ddots &{}\quad \vdots \\ c_{S,1-i} &{}\quad \cdots &{}\quad c_{S,N-i} \end{pmatrix}\\&\quad +\sigma ^{(8)}\beta _T \begin{pmatrix} t_{1} &{}\quad \cdots &{}\quad t_{N}\\ \vdots &{}\quad \ddots &{}\quad \vdots \\ t_{1} &{}\quad \cdots &{}\quad t_{N} \end{pmatrix} + \sigma ^{(9)}\beta _H\begin{pmatrix} h_{-24} &{}\quad \cdots &{}\quad h_{N-25}\\ \vdots &{}\quad \ddots &{}\quad \vdots \\ h_{-24} &{}\quad \cdots &{}\quad h_{N-25} \end{pmatrix}+\begin{pmatrix} \varepsilon _{11} &{}\quad \cdots &{}\quad \varepsilon _{1N}\\ \vdots &{}\quad \ddots &{}\quad \vdots \\ \varepsilon _{S1} &{}\quad \cdots &{}\quad \varepsilon _{SN} \end{pmatrix}. \end{aligned}$$

The indicator parameters $\sigma ^{(i)}$ take binary values and will serve to drop certain variables in the model specification (by value 0), whenever necessary. This model represents, perhaps, the simplest panel regression model in the following sense. The marginal effects of the lag incidence cases and those of the weather components could have been raised to matrices like in vector autoregression with exogenous variables (VAR-X) models^84^. Besides appending too many parameters (entries of the endogeneous matrices), which may lead to overfitting, VAR-X models also require all the explanatory variables to be covariance stationary (see^85^ for details), which is rarely the case for disease and weather data in the subtropics. As the only random spatial pattern was observed from the incidence data for almost all observations, no essential state-crossing marginal effects were expected. State-dependent marginal effects for the weather components were also not considered due to data aggregation and limitation, also to the intention to have unified marginal effects that work on the national level. Moreover, all lags smaller than the optimal values for the weather components were not considered for complexity reduction. For the reason of having straight-forward marginal effects, prior transformations were not applied to any of the variables. Despite its simplicity, the model (1) treats omitted variable bias by including individual-specific effects. These are the simplest terms assuming that the omitted variables only have constant effects on the daily COVID-19 cases in all the states. After all, the present study draws forth an outlook for compiling temperature and relative humidity data from all eligible stations as well as data of other confounding factors (e.g. other weather components, human mobility, employment opportunities, mapping of manufactures or public gatherings, etc) that not only add more explanatory variables but also clear up the heteroscedasticity issue.

### Model including incidence clustering

Previous studies based their investigation on asking which ranges of weather components correctly predict incidence cases. This study asks a slightly different question: which ranges of incidence cases are correctly predicted by the existing values of the weather components. The values that fail to predict certain incidence cases due to insignificance would deem dropping. In^68^, this clustering strategy was designed to eliminate the weather dependency on the zero incidence cases, handling the zero-inflation problem appropriately. In the context of COVID-19, some extreme cases might have never been related to weather, for example superspreading events^10–13^ and indoor aerosol transmission^8,9^. The basic aim of the clustering is then to correctly place the role of weather where it should have never predicted such events. The use of a transient function to replace this functionality was inapplicable to us, for which bias may arise from the functional choice and its related extension strategy for prediction.

The clustering idea departs from stratifying the incidence data into *M* clusters $(\Omega _{k})_{k=1}^M$ separated by barriers $\theta :=(\theta _{k})_{k=1}^{M-1}$. In the closed forms, the clusters are given by $\Omega _k=\{c:\max \{0,\theta _{k-1}\}\le c<\min \{\theta _k,\max _{i,j}c_{ij}\}\}$. Let us define the function $\delta _k(C;\theta ):=({\mathbbm {1}}_{\Omega _k}c_{ij})$, where ${\mathbbm {1}}_{\Omega _k}$ denotes the characteristic function, taking value 1 in case $c_{ij}$ belongs to $\Omega _k$ or 0 in case otherwise. Let us denote $P\circ Q=(p_{ij}q_{ij})$ as the Hadamard product between two matrices and define $T^k=T^k(\theta ):=\delta _k(C;\theta )\circ T$, $H^k=H^k(\theta ):=\delta _k(C;\theta )\circ H$. The latter return the original entries of the matrices *T*, *H* in case their pairing incidence cases belong to the corresponding cluster or 0 in case otherwise. Under this decomposition it always holds $\sum _kT^k=T$ and $\sum _kH^k=H$. Including clustering, a new model revises model (1) in the following fashion

2 $$\begin{aligned} C = \beta _0{\mathbbm {1}}_{S\times N} + \sigma ^{(0)}{\mathbbm {1}}_{N}^{\top }\otimes \beta _{\text {ind}}^{\top } + \sum _{i=1}^7\sigma ^{(i)}\beta _{-i}C_{-i}+\sum _{i=1}^3 \sigma ^{(7+i)}\beta ^i_TT^{(i)}+\sum _{i=1}^3 \sigma ^{(10+i)}\beta ^i_HH^{(i)}+\varepsilon . \end{aligned}$$

Here, the incidence data were classified into three clusters ($M=3$) on the basis of practicality to call for lower, middle, and upper cluster. In principle, the specification is not bound to such a small number as fitting would be better with more explanatory variables. However, questions regarding complexity and practical interpretations might arise when using a large number of clusters. On the present choice, when for instance $T^{(2)}$ has to be dropped due to insignificance, this simply means that the average temperature fails to predict incidence cases in the range defined by the middle cluster $\Omega _2$. This model then allows the lone cases to be “unexplained by temperature”.

The fact that $T^k$ and $H^k$ change with the lower and upper barrier $\theta =(\theta _{\text {l}},\theta _{\text {u}})$, so does the pooled estimator ${\hat{\beta }}={\hat{\beta }}(\theta )$ where $\beta =(\beta _0,\beta _{\text {ind}},\beta _{-1},\ldots ,\beta _{-7},\beta _{T}^1,\ldots ,\beta _{H}^3)$. Our aim is to find the optimal barriers such that the squared error between data $C=(c_{ij})$ and the model approximate $C[{\hat{\beta }}](\theta )$ achieves its minimum. Mathematically, the preceding statement translates to the following problem

3a $$\begin{aligned}&\!\min _{\theta }&\qquad&\sum _{i,j}\,(c_{ij}[{\hat{\beta }}](\theta )-c_{ij})^2 \end{aligned}$$

3b $$\begin{aligned}&\text {subject to}&\min _{i,j}c_{ij}\le \theta _{\text {l}}\le \theta _{\text {u}}\le \max _{i,j}c_{ij}. \end{aligned}$$

The pooled estimator ${\hat{\beta }}$ follows from the straightforward formula in terms of matrix inverse and multiplication involving explanatory and response variable.

## Results

### Case–weather cross-correlation and case-specific auto-correlation

Figure 4 represents the correlation coefficients on a moving window of 290 observations with time lags from 0 to 30 days for each state. Notice that the reported daily COVID-19 cases correlated negatively with the average temperature and positively with the average relative humidity. The magnitude of the correlation coefficient with average temperature shows decreasing trends with lag for all the states. With no lag introduced, the correlations are negative and significant for all the states (p-values from $6.27\times 10^{-34}$ to $1.17\times 10^{-15}$). Averaging the correlation coefficients throughout the states, the minimum of − 0.5223 was obtained. This negative correlation is comparable up to certain ranges of minimum, maximum and average temperature to the studies in Brazil (with both average ranging from 20.9 to 27 $^{\circ }$C and maximum temperature from 23.1 to 34.2 $^{\circ }$C in^57^ and with average temperature ranging from 16.8 to 27.4 $^{\circ }$C in^86^) as well as the data in 127 countries (with average temperature from − 17.8 to 42.9 $^{\circ }$C in^87^). In New York^88^, the correlation was positive and insignificant for average and minimum temperature but positive and insignificant for the maximum temperature. In Oslo, Norway^89^, the correlation was negative and insignificant for all maximum, minimum, and average temperature with 14 days time lag, but positive and significant correlation was obtained for normal temperature with 0, 5, 6, and 14 days lag. The temperature in Oslo ranged from − 7.5 to 21.9 $^{\circ }$C during the study period. COVID-19 cases in Russian Federation exhibited positive significant correlation with minimum (− 17.78 $^{\circ }$C to 8.89 $^{\circ }$C), maximum (0.56 $^{\circ }$C to 27.2 $^{\circ }$C) and average temperature (− 2.78 $^{\circ }$C to 16.1 $^{\circ }$C)^46^.

As far as relative humidity is concerned, it can be observed from Fig. 2 that its average varies from 39.38 to 93.53%. The best lag was found 25 days with the correlation coefficient value of 0.4194 from averaging throughout the states. With this lag, the correlations are positive and significant for all states (p-values from $2.98\times 10^{-18}$ to $1.92\times 10^{-8}$). For the relative humidity, different results preceded ours. A previous study in New York^88^ concluded that average relative humidity was insignificantly negatively correlated with the daily new cases. It was found that average humidity was significantly negatively correlated and relative humidity was insignificantly negatively correlated with the number of the ICU daily patients, according to data from Milan (14–100% for relative humidity, 1–23 $\text {g m}^{-3}$ for average humidity), Florence (10% to 100% for relative humidity, 1 to 23 $\text {g m}^{-3}$ for average humidity) and Trento (16–100% for relative humidity, 1 to 25 $\text {g m}^{-3}$ for average humidity) in Italy^90^. Data from Brazil ranging from 69.5 to 90.8% with no lag^50,57^ showed that the correlation was positive but not significant with minimum and maximum average humidity. Data from 127 countries^87^ led to the conclusion that the relative humidity was correlated negatively and insignificantly with daily new cases.

**Table 1:** Pearson and Spearman-rank correlation coefficients from the incidence data, rounded to two digits after comma.

$\rho$	lag-0	lag-1	lag-2	lag-3	lag-4	lag-5	lag-6	lag-7
lag-0	1							
lag-1	0.87, 0.83	1						
lag-2	0.83, 0.81	0.87, 0.83	1					
lag-3	0.80, 0.79	0.83, 0.81	0.87, 0.83	1				
lag-4	0.79, 0.79	0.81, 0.79	0.83, 0.79	0.87, 0.83	1			
lag-5	0.82, 0.80	0.80, 0.79	0.81, 0.79	0.83, 0.80	0.87, 0.83	1		
lag-6	0.87, 0.82	0.83, 0.80	0.80, 0.79	0.80, 0.79	0.83, 0.80	0.87, 0.83	1	
lag-7	0.89, 0.83	0.87, 0.82	0.83, 0.79	0.79, 0.78	0.80, 0.79	0.83, 0.80	0.87, 0.83	1

Table 1 shows the case-specific auto-correlations. Generally, Pearson is higher than Spearman-rank correlation coefficient. In addition, both Pearson and Spearman-rank correlation coefficient are significant with minimum 0.78 (p-values $\simeq 0$). From the column of lag-0, the auto-correlation generally swings from a large value at lag-1, then minima at either lag-3 or lag-4, to another large value at lag-7. The same behavior can be observed from the columns lag-1 until lag-3 where decrement rules out the first 4 lags and minima were found at either lag 3 or 4 days from the time series. This finding will set a basis for those in the panel regression models, as seen shortly.

### Spatial auto-correlation

**Figure 5:**
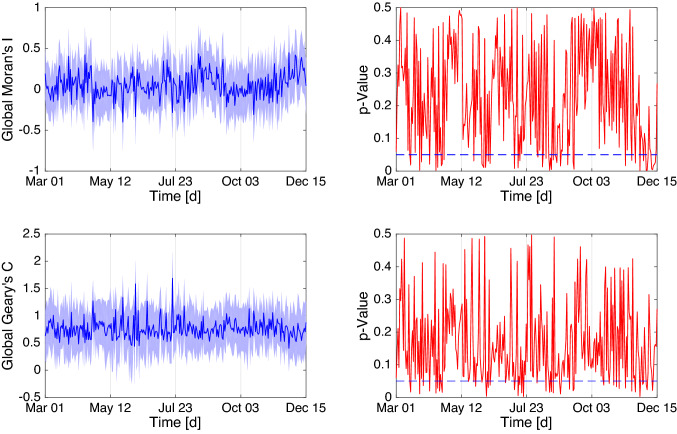
Global Moran’s ${\mathscr {I}}$ and Geary’s ${\mathscr {C}}$ computed on a daily basis together with the corresponding 95% confidence interval and p-Value (right) for significance. The blue dashed line represents the significance level $\alpha =0.05$.

Meanwhile previous studies much focused on aggregated data and variation of distances in the spatial weight matrix, this study computed the global Moran’s ${\mathscr {I}}$ and Geary’s ${\mathscr {C}}$ for all time to see how the spatial pattern changes seasonally since the earliest infection. The corresponding computation results together with the 95% confidence interval $[{\mathscr {I}}-1.93\sqrt{{\mathbb {V}}({\mathscr {I}})},I+1.93\sqrt{{\mathbb {V}}({\mathscr {I}})}]$ (respectively for ${\mathscr {C}}$) are presented in Fig. 5. Although the spatial pattern of the daily cases in all the states changes around with time, it is evident that randomness overwhelms the pattern for most of the time. The progression of p-values (especially below $\alpha$) indicates that, generally, no significant difference between Moran’s ${\mathscr {I}}$ and Geary’s ${\mathscr {C}}$ was observed except on the duration from November until mid of December where Geary’s ${\mathscr {C}}$ shows more locally clustered spatial pattern.

**Figure 6:**
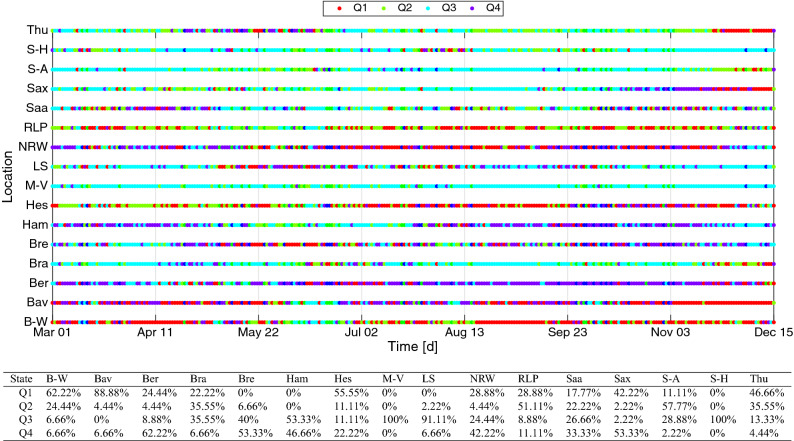
Classification into four quadrants $(\text {Q1},\,\text {Q2},\,\text {Q3},\,\text {Q4})$ equivalent to Moran’s scatter plot and the concurrence percentages from November 1 to December 15, 2020.

The Moran’s scatter plot for all the states in Germany was determined for all observations, see Fig. 6. For the sake of serial presentation, indexing the coordinates based on the quadrants is more favorable than plotting them. Overall, the results suggest that all the states show randomness with time in to which spatial pattern they belong. If one solely focuses on the recent observations (November 1 to December 15, 2020), then the following states have the tendency to occupy the following quadrants: Baden-Württemberg, Bavaria, Hesse, Thuringia (Q1); Brandenburg, Rhineland-Palatinate, Saxony-Anhalt (Q2); Hamburg, Mecklenburg-Vorpommern, Lower Saxony, Schleswig-Holstein (Q3); Berlin, Bremen, North Rhine-Westphalia, Saxony (Q4).

### Panel regression models

Variable choices for model specification were investigated. The criteria are based on not only fit and complexity (information-type criterion) but also insignificance, negative marginal effects, and multicollinearity driven by certain variables. For the fit and complexity, a minimal value of Bayesian Information Criterion $\text {BIC}=-2\log (L)+\log (N)\cdot k$^91^ was sought. The first term of this criterion expresses maximization over the likelihood function *L* generated from our model and the second term includes the observation size *N* as well as the number of parameters *k*. Unlike Akaike Information Criterion (AIC)^92^ that would have replaced $\log (N)$ by 2, BIC penalizes the number of parameters much more, especially for large observation sizes. Our study aims to drop certain variables toward cutting down BIC and amending insignificance as well as multicollinearity. The standard *t*-test was used for the significance test. Checking for multicollinearity follows from computing the Inverse Variance Inflation Factor (1/VIF) values for all explanatory variables except the constant. A 1/VIF measures one minus the coefficient of determination derived from an OLS-regression whereby the variable under test serves as the response while the others as the explanatory variables. In this sense, 1/VIF of a value smaller than the rule of thumb 0.1 shows multicollinearity driven by the tested variable^93^. In addition, the p-value of the *F*-statistic is monitored, which measures if the overall variables are simultaneously significant; of which smaller than $\alpha =0.05$ indicates that they are. Not only can the model be designated to be better than just a constant, but multicollinearity can also be diagnosed. Johnston in^94^ hinted the existence of multicollinearity as some p-values from *t*-tests are large while that from *F*-test is radically small, which agrees to the analytical investigation in^95,96^. Besides these aspects, if certain marginal effects would be consistent with our auto-correlation study were also checked. From Table 1, it is seen how cases in the past 7 days positively predict present cases with the least auto-correlations found from cases from the past 3 and 4 days. This led to dropping negative marginal effects corresponding to lag incidence cases that may occur due to a certain model specification.

**Figure 7:**
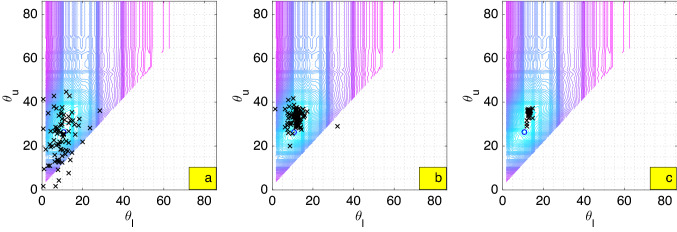
Computation of optimal barriers $(\theta _{\text {l}},\theta _{\text {u}})\approx (13.3645,36.0597)$ for the clustering. Blue circle encodes the optimal barriers found by the brute-force computations on the $50\times 50$ grid. The figures show the evolution of the locations of 100 players (black $\times$) converging to an optimal solution that does not overlap with the grid: (**a**) 5th iteration, (**b**) 10th iteration, (**c**) 20th iteration.

**Table 2:** Model specification under variable dropping. BIC values as well as corresponding issues leading to model exclusion are reported: S*i*, N*i*, M*i* stand for insignificance, negative marginal effect, and multicollinearity driven by the corresponding variable ordered by $\sigma ^{(i)}$, respectively.

**Model** (1)							
$\sigma ^{(i)}=0$ for *i*		0	0, 3	0, 4	0, 3, 4		
BIC	24,049.74	23,933.03	23,924.93	23,953.11	23,946.74		
Issue	S0, S3	S3, N4	N4	S3, N3			
**Model** (2)							
$\sigma ^{(i)}=0$ for *i*		0, 12	0, 12, 10	0, 12, 3	0, 12, 10, 3	0, 12, 10, 4	0, 12, 10, 3, 4
BIC	21,006.56	21,578	21,569.72	21,570.42	21,562.14	21,587.59	21,580.1
Issue	S0, S3, S10, M12	S3, S10	S3	S10	N4	N3, S3	

To deal with the model including incidence clustering (2), the computation of optimal lower and upper barrier $(\theta _{\text {l}},\theta _{\text {u}})$ as in (3) is necessary. The characteristic functions embedded in the objective function make the optimization problem non-smooth. The brute-force computations of the objective function in the upper-left triangle of the $50\times 50$ grid in the domain $[\min _{i,j}c_{ij},\max _{i,j}c_{ij}]^2$ and a PSO algorithm^68^ were put in comparison. From Fig. 7, PSO outperforms the brute-force computations in locating the optimal barriers that minimize the objective function, also in terms of computation time.

According to Table 2, the BIC value for the simple model (1) is relatively large, exacerbated by large degrees of freedom. The model including incidence clustering (2) gives the least BIC value due to a minimal likelihood function. Additionally, the insignificance of the entire individual-specific effects for both models was spotted. The rationale behind this can be connected to the fact that the entire profile of global and local spatial auto-correlation as well as the largest outbreak (“COVID-19 and weather situation in Germany” and “Spatial pattern”) show randomness for almost all observations. Therefore, no state was worth constant recruitment (weighting) for its neighborhood to show a consistent spatial pattern throughout the observations.

**Table 3:** Fitting results and diagnostics for the models (1) and (2). The abbreviations stand for the following: Val (value), StDev (standard deviation), *t* p-Val (p-value of the *t*-test for the variable significance), 1/VIF (Inverse Variance Inflation Factor for multicollinearity), *F* p-Val (p-value of the *F*-test for the overall variable significance), $\text {R}^2$ (coefficient of determination), Adj $\text {R}^2$ (adjusted coefficient of determination), D–W–H (p-value of Durbin–Wu–Hausman test for random-effects vs. fixed-effects estimator), Wo (p-value of Wooldridge test for the serial correlation), B–P LM (p-value of Breusch–Pagan test for random effect vs pooled estimator).

	Val	StDev	*t* p-Val	1/VIF	*F* p-Val	$\text {R}^2$	Adj $\text {R}^2$	D-W-H	Wo	B-P LM
Model (1)										
$\beta _0$	$-$.8742	.3787	.021		0	.8558	.8556	.5355	0	1
$\beta _{-1}$	0.1827	0.0142	0	0.1603						
$\beta _{-2}$	0.0984	0.0128	0	0.2011						
$\beta _{-5}$	0.0514	0.0135	0	0.2033						
$\beta _{-6}$	0.2736	0.0149	0	0.1716						
$\beta _{-7}$	0.4145	0.0155	0	0.1645						
$\beta _T$	$-$ 0.0295	0.0099	0.003	0.6390						
$\beta _H$	0.0246	0.0049	0	0.7054						
$\beta _0$	0.1949	0.0593	0.001		0	0.8544	0.8543	0.5556	0	1
$\beta _{-1}$	0.1918	0.0142	0	0.1619						
$\beta _{-2}$	0.1054	0.0128	0	0.2026						
$\beta _{-5}$	0.0604	0.0135	0	0.2056						
$\beta _{-6}$	0.2791	0.0150	0	0.1723						
$\beta _{-7}$	0.4166	0.0155	0	0.1646						
$\beta _0$	$-$ 2.0767	0.7908	0.009		0	0.3694	0.3691	1	0.0094	0
$\beta _T$	$-$ 0.5681	0.0185	0	0.7997						
$\beta _H$	0.2256	0.0097	0	0.7997						
Model (2)										
$\beta _0$	5.9089	0.2162	0		0	0.9148	0.9146	0.7646	0	1
$\beta _{-1}$	0.1378	0.0109	0	0.1590						
$\beta _{-2}$	0.0716	0.0098	0	0.1998						
$\beta _{-5}$	0.0337	0.0104	0	0.2031						
$\beta _{-6}$	0.1636	0.0117	0	0.1667						
$\beta _{-7}$	0.2866	0.0123	0	0.1543						
$\beta _T^l$	$-$ 0.1261	0.0076	0	0.4755						
$\beta _T^m$	0.3158	0.0224	0	0.4380						
$\beta _H^l$	$-$ 0.0528	0.0026	0	0.3687						
$\beta _H^u$	0.2033	0.0047	0	0.6981						
$\beta _0$	1.8381	0.3594	0		0	0.8682 (within) 0.9558 (between) 0.8692 (overall)		0	0.0097	0
$\beta _T^l$	$-$ 0.2088	0.0092	0	0.4927						
$\beta _T^2$	$-$ 0.1010	0.0292	0.001	0.3878						
$\beta _T^3$	$-$ 0.6897	0.1037	0	0.4472						
$\beta _H^l$	0.0524	0.0046	0	0.1785						
$\beta _H^2$	0.2627	0.0051	0	0.1243						
$\beta _H^3$	0.5608	0.0085	0	0.3258						

Post-estimation diagnostics for all the models including those investigated during model specification were performed. Additional to the models including lag incidence cases and weather components, this study considered the models where either of these entities is present. The fitting results are presented in Table 3. For straightforward marginal effects and computation of optimal barries, the pooled estimator was considered subject to its inefficiency. The test was conducted via the comparison between fixed-effects and random-effects estimator and that between random-effects and pooled estimator. To the former, the two estimators were compared using Durbin–Wu–Hausman test^97,98^, where the fixed-effects estimator is assumed to be consistent, and the random-effects estimator is efficient and assumed to follow a normal distribution. The null hypothesis suggests that the random-effects estimator is a consistent estimator regardless of the size of the data. According to Table 3, the p-value corresponding to the statistic greater than $\alpha =0.05$ indicates that the random-effects estimator is equally consistent as the fixed-effects estimator. The two estimators for all presented models confirm equivalence except for model (2) where only weather components are present. For this case, the fixed-effects estimator was kept to handle consistency and panel effect. To the latter, Breusch–Pagan Lagrange Multiplier test was done under no panel effect as the null hypothesis^99^, i.e., the model under the random-effects estimator returns zero variance in the state-dependent errors. Apparently, no panel effect was observed for all models except for those that include only weather components, in which case either random-effects or fixed-effects estimator is preferable. The inefficiency of the presented pooled, random-effects, and fixed-effects estimator is confirmed as serial correlation in all the state-dependent errors occurred. Wooldridge test^100^ showed this. Therefore, a caveat remains for all models that their standard deviations of the coefficients are smaller and $\text {R}^2$’s are larger than they should be. After all, the pooled estimator is always consistent, even for a relatively small data size. As final practical remarks from the models, all the lag incidence cases give the waving effects in terms of lag where the cases 5 days and 7 days from presently predict the present cases the least and the most, respectively. Keeping the lag incidence cases, the weather components from model (1) give a consistent prediction with that from the cross-correlation study. Together with clustering, the marginal effects of weather were corrected for model (2). It was observed that temperature fails to predict cases in the upper cluster while relative humidity fails to cases in the middle cluster. Temperature seems to give a larger positive marginal effect for the middle cluster while relative humidity a negative smaller marginal effect for the lower cluster.

**Figure 8:**
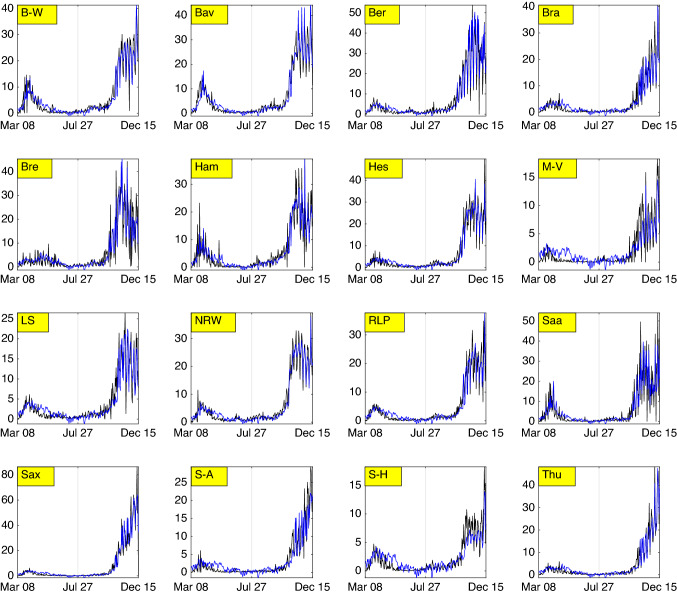
Fitting result (in blue) for the model including incidence clustering.

As far as predictive performance is concerned, several findings can be highlighted. As the larger models exhibit no more issues with insignificance and multicollinearity, neither do the smaller models. For the model variant (1), the smaller models gain $\text {R}^2\approx 0.8544$, $\text {BIC}\approx 23{,}972.15$ (only lag incidence cases) and $\text {R}^2\approx 0.3694$, $\text {BIC}\approx 30585.35$ (only weather components), respectively. Meanwhile the model including only weather components shows the poorest performance; its BIC value is also radically larger than that of the model including only lag incidence cases. For the model (1), the impact of weather is rather small, as the decrease of temperature from a reference value e.g. $T\approx 20\,^\circ \text {C}$ to $T\approx 10\,^\circ \text {C}$ (i.e. by $50\%$) is associated to the increase of COVID-19 cases for all states from e.g. $C\approx 20$ by $(|\beta _T|10/20)\cdot 100\%\approx 1.475\%$. When the lag incidence cases were dropped, the increase changes to $(0.5681\cdot 10/20)\cdot 100\%\approx 28\%$. Moreover, the increase of relative humidity from 60 to 80% (by 33%) is associated to the increase of the cases from $C\approx 20$ by 2.46% (with lag incidence cases) and 22.56% (without lag incidence cases). The overall impression indicates the superiority of the model with only lag incidence cases when one designates fit to significantly matter than the number of parameters. For the model including incidence clustering (2), a different profile was obtained when only using non-dropped weather components: $\text {R}^2\approx 0.7948$, $\text {BIC}\approx 25517.61$. Here, a significant improvement under incidence clustering becomes evident. Surprisingly, the model including the entire weather components even outperforms that including only lag incidence cases by fit and complexity: $\text {R}^2\approx 0.8692$, $\text {BIC}\approx 23494.94$. All marginal effects corresponding to the temperature matrices are negative, and those corresponding to the relative humidity matrices are positive. It was observed that the temperature returns the smallest marginal effect on the COVID-19 cases in the middle cluster and relative humidity in the lower cluster. Besides the significance of the marginal effects, even no multicollinearity was observed. Apart from this, when the predictive ability is evaluated by $\text {R}^2$ and $\text {BIC}$ amending multicollinearity and inconsistent predictors, it is still argued that combining lag incidence cases and weather components serve as the best models as presented in Table 3. The corresponding graphical fitting can be seen in Fig. 8.

## Discussion

In this study, lags from the cross-correlation between average temperature and relative humidity were extracted to synthesize suitable variables in the regression models. Additionally, case-specific auto-correlation supports the model specification where lag-3 and lag-4 incidence would rather be insignificant predictors for the present incidence. Spatial auto-correlation using global Moran’s ${\mathscr {I}}$ and global Geary’s ${\mathscr {C}}$ was investigated in the framework of analyzing the spatial effect in COVID-19 transmission. The global measures indicate random spatial patterns most of the time, except there were either local clusters or dispersion in recent observations from November 1 to December 15, 2020. Moran’s scatter plot was then used to disclose the local behavior of the spatial pattern. The result shows that the distribution of the hot spots and cold spots generally changed with time. The random spatial pattern justifies the model specification where the individual- or state-specific effects that would have served to endow specific states with constant weighting factors, were dropped.

In the simple random-effects model, the average temperature and lag relative humidity were shown to affect the incidence significantly, however, the resulting coefficient of determination is comparably much smaller than whenever only lag incidence cases were used; panel effect also raises in the former case. For the reason of placing the correct role of weather in predicting certain ranges of incidence, the weather components were grouped with the aid of a clustering strategy. The new clustering-integrated model accompanied by optimal barriers shows good agreement with the data whereby weather components outperform lag incidence cases in the prediction. On this matter, the fixed-effects estimator was the only presumably consistent estimator that also tackles the panel effect. For all models, it was observed that every explanatory variable competes against the others to be a significant predictor. Therefore, model choice together with its consequences (marginal effects), depend entirely on the decision-maker. Marginal effects can be guidance when a model is chosen a priori. When $\text {R}^2$ and BIC matter a lot, our recommendation is to opt for the clustering-integrated model with lag incidence cases and lag weather components. There it was found that temperature and relative humidity have negative, relatively small marginal effects on the cases in the lower cluster (below 13 cases per 100,000 inhabitants); the temperature has a large positive marginal effect on the cases in the middle cluster (between 13 and 36 cases per 100,000 inhabitants) and no marginal effect on the upper cluster (above 36 cases per 100,000 inhabitants); relative humidity has a large positive marginal effect on the upper cluster but none on the middle cluster. The clustering-integrated model with only weather components is recommended when weather receives more privilege than lag incidence cases. Our result is consistent with the cross-correlation study that temperature has negative marginal effects while relative humidity has positive marginal effects on the incidence in all clusters. The middle cluster receives the smallest marginal effect from temperature and the lower cluster from relative humidity. This hints physical consequences that temperature can only predict incidence cases during hot (summer) and cold season (winter), where cases clearly distinguish against each other from the data, not during transitional seasons (spring and fall). Relative humidity, on the other hand, is less likely to predict sinking cases during the hot season.

## Conclusion

This study focused on the interrelationship between two weather components overlapping in many previous studies (average temperature and relative humidity) and COVID-19 incidence in Germany. Cross-correlation, case-specific auto-correlation, and spatial auto-correlation analysis were done to determine suitable variables and to explain the negligible panel effect in the panel random-effects models. In addition, the findings from the spatial auto-correlation provide the placement of the 16 states in the four quadrants from Moran’s scatter plot and appropriate policy regarding traveling restrictions. The increasing demand for confounding factors to explain various incidence levels has been neutralized by the aid of incidence clustering. This strategy supports the idea of considering only certain hypothetical factors predicting COVID-19 incidence and general regression modeling wherein explanatory variables are limited. This localization of incidence that is correctly predicted by the two weather components has profound implications for public health authorities. The modeling does not only determine the extent of the prediction via marginal effects but also paves the way for precautionary actions amidst upcoming weather.

## Data Availability

All the data sources have been included in “COVID-19 and weather situation in Germany”.
