# Publisher Correction: Interrelationship between daily COVID-19 cases and average temperature as well as relative humidity in Germany

**DOI:** 10.1038/s41598-021-97830-2

**Published:** 2021-09-08

**Authors:** Naleen Chaminda Ganegoda, Karunia Putra Wijaya, Miracle Amadi, K. K. W. Hasitha Erandi, Dipo Aldila

**Affiliations:** 1 Department of Mathematics, University of Sri Jayewardenepura, Nugegoda, 10250 Sri Lanka; 2 Mathematical Institute, University of Koblenz, 56070 Koblenz, Germany; 3 Department of Mathematics and Physics, Lappeenranta University of Technology, 53851 Lappeenranta, Finland; 4 Department of Mathematics, University of Colombo, Colombo, 00300 Sri Lanka; 5 Department of Mathematics, Universitas Indonesia, Depok, 16424 Indonesia

Correction to: *Scientific Reports* 10.1038/s41598-021-90873-5, published online 28 May 2021

The original PDF version of this Article contained a repeated error where the symbol “” was incorrectly given as “” in Equations 1 and 2, and subheadings “Simple case-weather relation” and “Model including incidence clustering”.

This error has now been corrected in the PDF version of the Article; the HTML version was correct from the time of publication.

